# A silent gradient axis for soundless spatial encoding to enable fast and quiet brain imaging

**DOI:** 10.1002/mrm.29010

**Published:** 2021-09-21

**Authors:** Edwin Versteeg, Dennis W. J. Klomp, Jeroen C. W. Siero

**Affiliations:** ^1^ Department of Radiology University Medical Center Utrecht Utrecht The Netherlands; ^2^ Spinoza Center for Neuroimaging Amsterdam Netherlands

**Keywords:** gradient coil, gradient insert, high gradient slew rate, magnetic resonance imaging, peripheral nerve stimulation, plug‐and‐play, quiet, silent

## Abstract

**Purpose:**

A novel silent imaging method is proposed that combines a gradient insert oscillating at the inaudible frequency 20 kHz with slew rate‐limited gradient waveforms to form a silent gradient axis that enable quiet and fast imaging.

**Methods:**

The gradient insert consisted of a plug‐and‐play (45 kg) single axis *z*‐gradient, which operated as an additional fourth gradient axis. This insert was made resonant using capacitors and combined with an audio amplifier to allow for operation at 20 kHz. The gradient field was characterized using field measurements and the physiological effects of operating a gradient field at 20 kHz were explored using peripheral nerve stimulation experiments, tissue heating simulations and sound measurements. The imaging sequence consisted of a modified gradient‐echo sequence which fills *k*‐space in readout lanes with a width proportional to the oscillating gradient amplitude. The feasibility of the method was demonstrated in‐vivo using 2D and 3D gradient echo (GRE) sequences which were reconstructed using a conjugate‐gradient SENSE reconstruction.

**Results:**

Field measurements yielded a maximum gradient amplitude and slew rate of 40.8 mT/m and 5178T/m/s at 20 kHz. Physiological effects such as peripheral nerve stimulation and tissue heating were found not to be limiting at this amplitude and slew rate. For a 3D GRE sequence, a maximum sound level of 85 db(A) was measured during scanning. Imaging experiments using the silent gradient axis produced artifact free images while also featuring a 5.3‐fold shorter scan time than a fully sampled acquisition.

**Conclusion:**

A silent gradient axis provides a novel pathway to fast and quiet brain imaging.

## 1 INTRODUCTION

Acoustic noise during an MR exam can cause substantial discomfort in subjects and pose a potential health risk due to the high sound pressure levels reached (>100 dB) during gradient‐intensive sequences.^1^, ^2^ This acoustic noise originates from the interaction between the rapidly switching currents in the gradient coil and the main magnetic field through the Lorentz force, causing the gradient coils to vibrate. For reducing acoustic noise, a favorite approach is to reduce the gradient switching rate, which is done by reduced slew rates, smooth waveforms or alternative encoding schemes (RUFIS), albeit at the cost of scan time.^3^, ^4^, ^5^, ^6^

Alternatively, one could exploit the limits of human sound perception to decrease the sound in MRI exams. Perceptible sounds span a frequency range from 20 to 20 000 Hz in young healthy people. The upper limit of this range generally decreases with age as well as the sensitivity to sound. As sound produced outside this frequency range will not be heard, a gradient coil operating near or at the upper limit would be inaudible.

For whole‐body gradient coils using trapezoidal gradient waveforms, the slew rate (SR) is limited to around 200 T/m/s by human physiology in the form of peripheral nerve stimulation (PNS).^7^, ^8^, ^9^ The severity and likelihood of inducing PNS are proportional to the peak magnetic field change per unit time induced by the gradient coil, which increases at higher slew rates.^10^, ^11^ However, PNS does not only depend on the gradient slew rate but also on the frequency of the applied gradient waveform. Studies using magnetic stimulation on arms and legs have shown that PNS becomes independent of switching rate for frequencies from 10 up to 100 kHz and that PNS thresholds even increase for even higher frequencies (>100 kHz).^12^, ^13^ Specifically, for switching at a high frequency (>10 kHz), the peak magnetic field needed to induce PNS was found to approach a constant value determined by the imaged body part. Consequently, while maintaining the absolute peak magnetic field change under a certain level, one can increase the switching frequency by orders of magnitude without causing PNS.

One way to limit the peak magnetic field change is to use a local gradient coil or gradient insert. Such a gradient insert is usually designed for a specific anatomy, eg, the head, which allows for a smaller coil size when compared to their whole‐body counterparts. The smaller coil size confines the linear field gradient to a smaller area, which yields lower peak magnetic field changes and limits peak electric fields by exposing a smaller part of the body when compared to a larger coil for a given field gradient. As a consequence, these gradient inserts allow for faster switching without inducing PNS.^8^, ^14^, ^15^, ^16^

The first gradients inserts (early 1990s) were small head‐coil sized coils that aimed to overcome the hardware (not PNS) limited gradient performance of whole‐body gradients at the time (G = 10 mT/m, SR = 20 T/m/s).^14^, ^17^ Current whole‐body gradients feature an order of magnitude higher gradient performance (G = 40 mT/m, SR = 200 T/m/s), which is made possible by the use of pulse‐width modulated (PWM) gradient amplifiers that can supply the high voltages and currents necessary (up to 1 kA and 2 kV).^18^ However, their gradient performance is limited by PNS. State‐of‐the‐art gradient inserts, therefore, try to limit PNS while achieving higher gradient performance. This is possible using improved gradient designs methods^19^, ^20^, ^21^, ^22^ that allow for the gradient design to incorporate features such as active shielding to mitigate eddy currents, cost function‐guided optimized coil winding placement balancing linearity and slew rate, water cooling to reduce heating and asymmetric designs to improve subject positioning.^15^, ^23^, ^24^, ^25^ In practice, these state‐of‐the‐art gradient inserts are used for high‐resolution fMRI and diffusion MRI and can reach gradient strengths of 200 mT/m and slew rates up to 1300 T/m/s.

The slew rate of a sinusoidal gradient waveform at a fixed gradient amplitude scales linearly with the oscillation frequency. At inaudible frequencies (>20 kHz), this means that high slew rates on the order of 5000 T/m/s are needed to reach the whole‐body gradient performance of a commonly used maximum gradient amplitude of 40 mT/m. Unfortunately, these high slew rates are not possible with current gradient inserts due to a combination of relatively high coil inductance and insufficient voltage of the gradient amplifier to drive the coil at 20 kHz. A solution for this can be found in early echo planar imaging (EPI)‐experiments. Here, resonant gradient chains were used to get high slew rates with low‐voltage.^26^, ^27^ This resonant behavior was obtained by switching one or multiple capacitors in series with the gradient coil and enabled efficient power transfer between the amplifier and gradient coil at a specific frequency. Importantly, this principle will also work for inaudible frequencies as long as the used amplifier can deliver power at frequencies above 20 kHz.

In this work, we present a novel silent imaging method that exploits the limits of human sound perception. Here, we propose to drive the most dominant gradient axis, in terms of sound production, at a frequency above 20 kHz by making the gradient insert coil resonant and excluding close proximity radiofrequency (RF) shields to minimize short term eddy currents. When operating the gradient insert in conjunction with the body gradients, we could maintain default functionality while facilitating 20 kHz readout gradients, which yields a silent gradient axis without PNS. We aim to use this setup to reduce sound in MRI while sacrificing no scan time, which is achieved by using the silent gradient axis as an additional spatial encoding axis in combination with a conventional MR sound reduction method that limits the slew rate of the imaging gradients. The feasibility of our novel silent imaging method will be demonstrated by the characterization of the gradient performance of our silent gradient axis, the investigation of biophysical effects such as PNS, tissue heating and sound at 20 kHz and the introduction of an imaging sequence and reconstruction framework incorporating the silent gradient axis.

## 2 METHODS

### 2.1 Hardware

The silent gradient axis consisted of a single‐axis lightweight (45 kg) gradient with a length of 45 cm, an outer diameter of 39.8 cm, an inner diameter of 28 cm, a 16 cm linear region (with a maximum deviation of 5% from a perfectly linear gradient field, calculated using Equation 7 in Ref. [28]), 113 µH inductance at 1 kHz and an efficiency of 0.32 mT/m/A, which operated in the *z*‐direction (Futura, Heerhugowaard, The Netherlands). Detailed maps of the field linearity of the coil can be found in our paper that describes the specifications of the gradient coil.^24^ The gradient insert could produce a maximum gradient amplitude of 200 mT/m and slew rate of 1300 T/m/s when paired with a conventional gradient amplifier (990 V, 630 A), was not actively shielded and was designed to be plug‐and play, which means it can be easily (de‐)installed between scan sessions.^24^ In this work, the gradient insert was used in a 7T scanner (Achieva, Philips, Best, The Netherlands) and included a built‐in radiofrequency transmit coil tuned to the proton resonance frequency (298 MHz) at 7T. Importantly, this transmit coil did not feature an RF shield as the close proximity to the gradient conductors, and the high switching rate of the gradient can cause excessive heating in the RF shield and reduce the efficiency of generating the gradient field.

The gradient insert was paired with an audio amplifier (k20, Powersoft, Italy) that can produce 18 kW peak power. The output voltage (450 V_peak_) of this audio amplifier is too low to directly drive the gradient insert at 20 kHz despite the low inductance of the insert (L = 106 µH at 20 kHz). Consequently, tuning and matching capacitors (*C*_tune_ = 470nF and *C*_match_ = 270 nF Snubber, Vishay, Selb, Germany) were added to the gradient insert to allow for optimal power transfer by impedance matching the coil to 4 Ω. Here, the capacitors were mounted on the cable‐side of the gradient connector, which means that gradient insert could still be operated in a non‐resonant mode by using a different gradient cable. Importantly, the audio amplifier did not feature any active water cooling (only air cooling). Consequently, the gradient amplitudes and repetition times used for imaging sequences were chosen to avoid overheating the amplifier.

**FIGURE 1:**
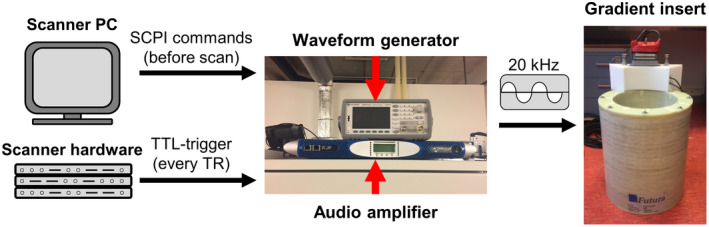
Schematic overview of the hardware setup used to control the silent gradient axis. Here, the scanner PC initializes the waveform generator before the scan using a SCPI protocol (Standard Commands for Programmable Instruments) that sends the requested gradient waveform, amplitude, frequency, duration and timing. During the scan the MRI scanner hardware controls the waveform generator by sending out a trigger pulse each repetition time. The output from the waveform generator is amplified by the audio amplifier which drives the resonant gradient insert at 20 kHz

A dedicated waveform generator (Keysight, USA) provided the input waveform to the audio amplifier. Before starting a scan, this waveform generator communicated with the scanner software through a python script (Python Software Foundation, https://www.python.org/) running on the scanner computer, which sends the waveform parameters; gradient waveform, amplitude, frequency, duration, and timing using a SCPI (Standard Commands for Programmable Instruments) protocol over a LAN‐connection. During scanning, the waveform generator was controlled through a TTL‐trigger pulse that was generated by the MRI scanner hardware for each repetition time. By using the dedicated waveform generator in this manner, the silent gradient axis can operate as an independent fourth gradient axis. An overview of the setup can be found in Figure 1.

### 2.2 Field measurements

The gradient field produced by the silent gradient axis was measured using a dynamic field camera system (Skope, Switzerland) consisting of 16 radiofrequency probes filled with liquids at fixed relative positions that detect local magnetic field changes from the phase of the induced MR signal. The field camera and gradient insert were placed in the MR system and controlled using a TTL‐trigger pulse. For each measurement, the silent gradient was pulsed for 24 ms using the maximum output of the amplifier and the field evolution was sampled for 100 ms at 1 MHz. Gradient fields were obtained from the first‐order spatial field component fitted by the field camera system, and slew rates were calculated by taking the time‐derivative of the measured gradient waveform.

### 2.3 Physiological effects

#### 2.3.1 Peripheral nerve stimulation

The PNS characteristics of the coil were assessed in four healthy volunteers (3 male/1 female 29–45 y). The slew rate dependency for a switching rate of 20 kHz was tested by presenting the subjects with a random sequence of pulse trains each with a different amplitude (ranging from 7.5 to 40 mT/m or in terms of slew rate 952–5076T/m/s). Four pulse‐trains of 50 ms were applied at each amplitude, with a pause of 1 s between pulse‐trains. The subjects were instructed to press an alarm button when they experienced PNS sensations, after which they indicated the location and rated the intensity of the sensations on a scale of 1–5 (1 = very mild, 5 = painful). Informed consent was given by all volunteers in accordance with the local Institutional Review Board. All subjects are MRI experts and were familiar with PNS.

#### 2.3.2 Specific absorption rate simulations

PNS is not the only physiological effect of fast switching magnetic fields. Such high frequency magnetic field might also induce tissue heating, which is a phenomenon already well known from radiofrequency coils in MRI, switched in the MHz range,^29^ and magnetic particle imaging, switched in the 100 kHz range.^30^ To assess this, simulations of the specific absorption rate (SAR) were performed in an electromagnetic finite‐difference time‐domain (FDTD) solver (Sim4Life, Zurich, Switzerland) on a detailed human body model (Duke, IT’IS Foundation, Zurich, Switzerland). In these simulations, the head of the human body model was positioned inside a model of the gradient insert, which was driven at its maximum gradient amplitude of 40 mT/m, at 20 kHz and at a 100% duty cycle. The obtained SAR‐values were compared to the local SAR head limit of 10 W/kg specified by the International Electrotechnical Commission (IEC).^31^

#### 2.3.3 Sound measurements

Sound produced by the silent gradient axis at 20 kHz cannot be heard, however, there are still exposure limits comparable to audible sounds.^32^, ^33^, ^34^ Therefore, sound measurements were performed using a condenser microphone (Behringer ECM8000) placed in the gradient insert and positioned to mimic the position of the ear during scanning. This microphone featured a flat response extending up to around 22 kHz and could, thus, measure sound at 20 kHz.

To estimate the sound level at 20 kHz, the sound produced by the gradient insert at 20 kHz was compared to the sound produced by the same insert at a more traditional 2 kHz switching rate. Here, the gradient insert was driven at 30 mT/m at both frequencies and a conventional gradient amplifier (NG500, Prodrive, Eindhoven, NL) was used to drive the gradient insert at 2 kHz. For these measurements, the gradient insert was pulsed for 100 ms and power spectra of the audio signal were calculated.

The sound level was also assessed during the scan we used for the 3D imaging experiments performed with the silent gradient axis. The microphone was calibrated using a 94 dB noise source (Bruel & Kjaer sound level calibrator type 4231). The audio data was processed in MATLAB, where exponential filtering and A‐weighting was applied to correspond to the fast response setting and output of a sound level meter.

### 2.4 Imaging sequence

The spatial encoding capabilities of an oscillating gradient is proportional to the gradient amplitude and inversely proportional to the oscillation frequency. Here, the resolution encoded in a half period (i.e., 25 μs for 20 kHz) is given by:

(1) $$\Delta x=\frac{\pi f}{\frac{\gamma }{2\pi }G}$$

In Equation (1), Δx is the resolution in the direction of the oscillating gradient in meters, γ is the gyromagnetic ratio in rad/s/T, G is the oscillating gradient strength in T/m and *f* is the oscillation frequency in Hz. For 1 mm resolution at 20 kHz, this equation shows us that a gradient amplitude of 1.47 T/m is needed for single‐shot encoding, which is not practically feasible when considering both the limited power available from the audio amplifier and possibly physiological effects like PNS. Therefore, we have implemented a segmented readout, which combines conventional cartesian encoding with an extra silently oscillating readout gradient. This is similar to acceleration methods like bunched phase‐encoding and Wave‐CAIPI, which also play out oscillating gradient during the readout albeit at an order of magnitude lower (and audible) frequency.^35^, ^36^

**FIGURE 2:**
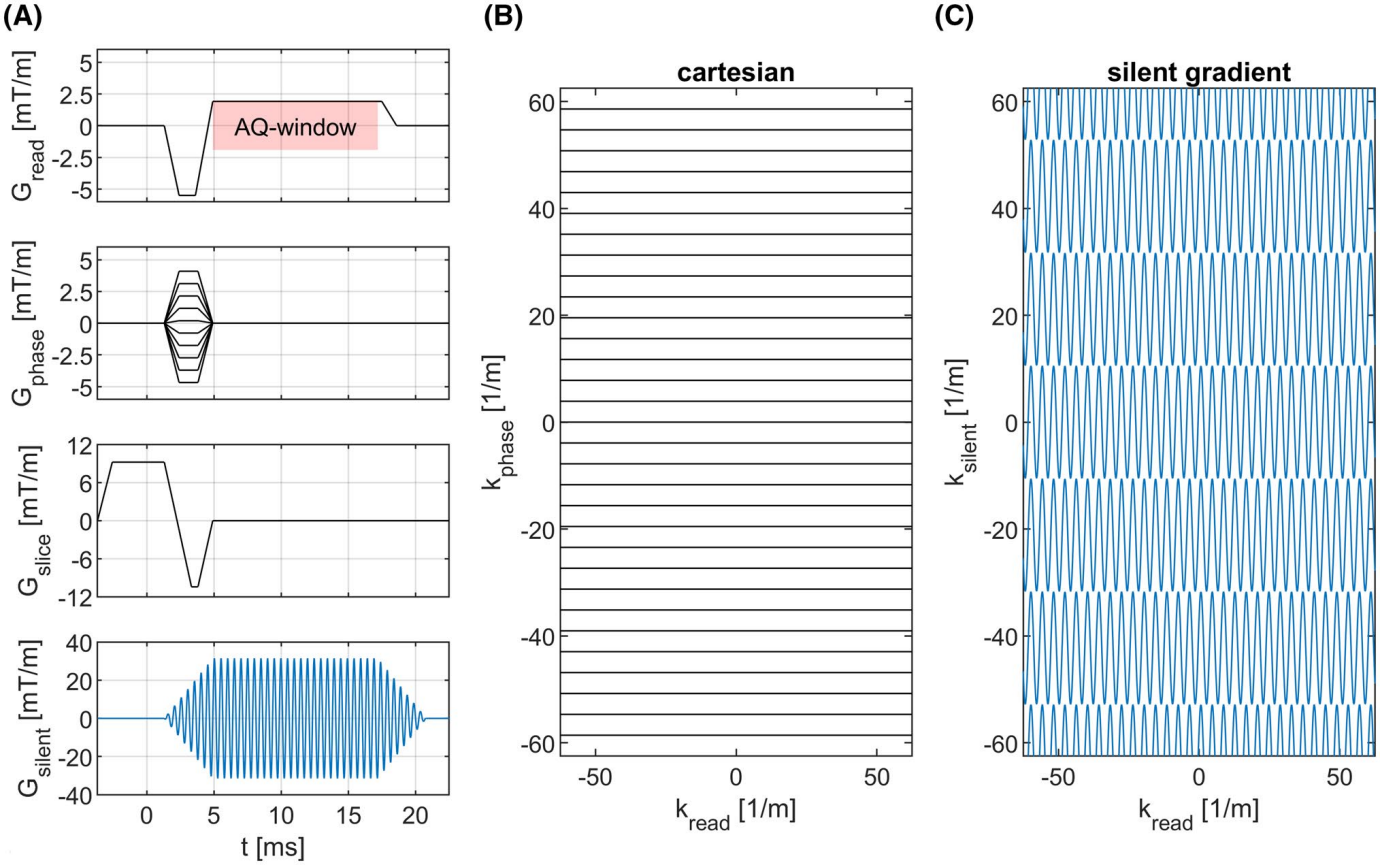
A, Sequence diagram of a 2D GRE sequence incorporating the silent gradient. Here, the black lines represent gradients that could also be used for cartesian imaging with in blue the oscillating silent gradient (displayed at a lower frequency). The red box represents the acquisition window. B, The *k*‐space filling of a conventional cartesian sequence and C, the sequence with the silent gradient axis. This highlights the wider lanes filled in *k*‐space when using the silent gradient axis for encoding

The segmented readout consists of a conventional cartesian gradient‐echo sequence with a modified readout gradient and an extra oscillating readout gradient. The sequence diagram is shown in Figure 2A. The modified readout gradient amplitude was chosen to satisfy the Nyquist criterion and was determined by the silent gradient oscillation frequency and the field of view (FOV). The gradient strength of this modified readout gradient was calculated as:

(2) $$G_{\text{read}}=\frac{f}{FOV_{\text{read}}\frac{\gamma }{2\pi }}$$

Here, $G_{\text{read}}$ is the readout gradient strength in mT/m, f is the oscillation frequency of the silent gradient in Hz, γ is the hydrogen gyromagnetic ratio in rad/s/T and $FOV_{\text{read}}$ is readout FOV in meters.

The oscillating silent gradient is played out simultaneously with the modified readout gradient. As a result of this extra spatial encoding, *k*‐space is now filled in readout lanes instead of lines, which means that larger phase‐encoding steps are done in the direction of the oscillating gradient. Here, each readout lane has a lane width proportional to the gradient amplitude of the silent gradient axis, which can be calculated by inverting Equation (1):

(3) $$\Delta k_{\text{silent}}=\frac{\frac{\gamma }{2\pi }G}{\pi f}$$

In Equation (3), $\Delta k_{\text{silent}}$ is the lane width in the direction of the oscillating gradient in meters, γ is the gyromagnetic ratio in rad/s/T, G is the oscillating gradient strength in T/m and f is the oscillation frequency in Hz. A comparison of the *k*‐space trajectory obtained with the segmented readout and a conventional cartesian readout is given in Figure 2B.

The extra spatial encoding provided by the oscillating gradient also allows us to perform sound reduction by limiting the slew rate of the whole‐body gradient used for the slice/slab‐selection, phase‐encoding and modified readout. Conventionally, this slower switching of gradients results in longer repetition times and, consequently, longer scan times. For the silent gradient axis, this increase in repetition time can be more than compensated, as fewer phase‐encoding steps and, thus, fewer total repetitions are needed due to the extra encoding provided by the readout lanes.

### 2.5 Imaging experiments

Imaging was performed on a 7T scanner (Achieva, Philips, Best, The Netherlands) with a 32‐channel receive coil (Nova Medical, Wilmington, MA, USA) which was positioned in the gradient insert. Both 2D and 3D images were acquired to test the feasibility of imaging with the silent gradient axis. These scans featured a long repetition time and an oscillating gradient amplitude of 31.5 mT/m to limit the heating of the audio amplifier.

For the 2D images the following sequence parameters were used: in‐plane resolution = 1 × 1 mm^2^, FOV = 256 × 256 mm^2^, slice thickness = 2 mm, flip angle = 22°, readout bandwidth = 81 Hz/pixel, 24‐fold oversampling in the readout direction (*f*_sampling_ = 500 kHz), TR = 62 ms, and TE = 11.2 ms The silent gradient axis amplitude of 31.5 mT/m yielded phase‐encoding steps of Δ*k*_silent_ = 21 m^−1^, which corresponded to a 5.3‐fold gain in scan time compared to a fully sampled acquisition. To investigate the effect of the silent gradient axis on the images, the 2D gradient echo (GRE) ‐acquisition was repeated once with smaller phase‐encoding steps (Δ*k*_silent_ = 1/FOV) and once without the silent gradient axis enabled. Except for scan time, imaging parameters were kept constant in these acquisitions.

The 3D acquisition featured the following sequence parameters: resolution = 1 × 1 × 2 mm^3^, FOV = 256 × 256 × 72 mm^3^, flip angle =22 degrees, readout bandwidth = 81 Hz/pixel, 24‐fold oversampling (*f*_sampling_ = 500 kHz), TR = 62 ms, and TE = 11.2 ms The same 3D GRE scan was also obtained without the 20kHz readout gradient, but only using the conventional body gradients. Additionally, a fully sampled scan without the encoding from the silent gradient axis was acquired which took 5.3‐fold longer to complete. Informed consent was given by all volunteers in accordance with the local Institutional Review Board. Importantly, hearing protection was used in all imaging scans.

### 2.6 Reconstruction

The reconstruction of the aforementioned imaging experiments was performed offline in MATLAB (Mathworks, Natick, MA, USA). A generalized conjugate‐gradient (CG) SENSE algorithm was used to perform the iterative reconstruction.^37^ The inputs to this algorithm were the raw data from scanning, the spatial encoding trajectory and a coil sensitivity map. Here, the field measurements provided the spatial encoding trajectory for the oscillating silent gradient and was used for temporal alignment between silent gradient axis and whole‐body gradients. *K*‐space sample density compensation was performed to compensate for the variations resulting from the rapidly oscillating gradient. The coil sensitivity map was obtained using an additional scan and was gridded to the same resolution as the target image. A non‐uniform Fourier transform (GPUNUFFT^38^) was used to transform between *k*‐space and image‐space.

## 3 RESULTS

### 3.1 Field measurements

**FIGURE 3:**
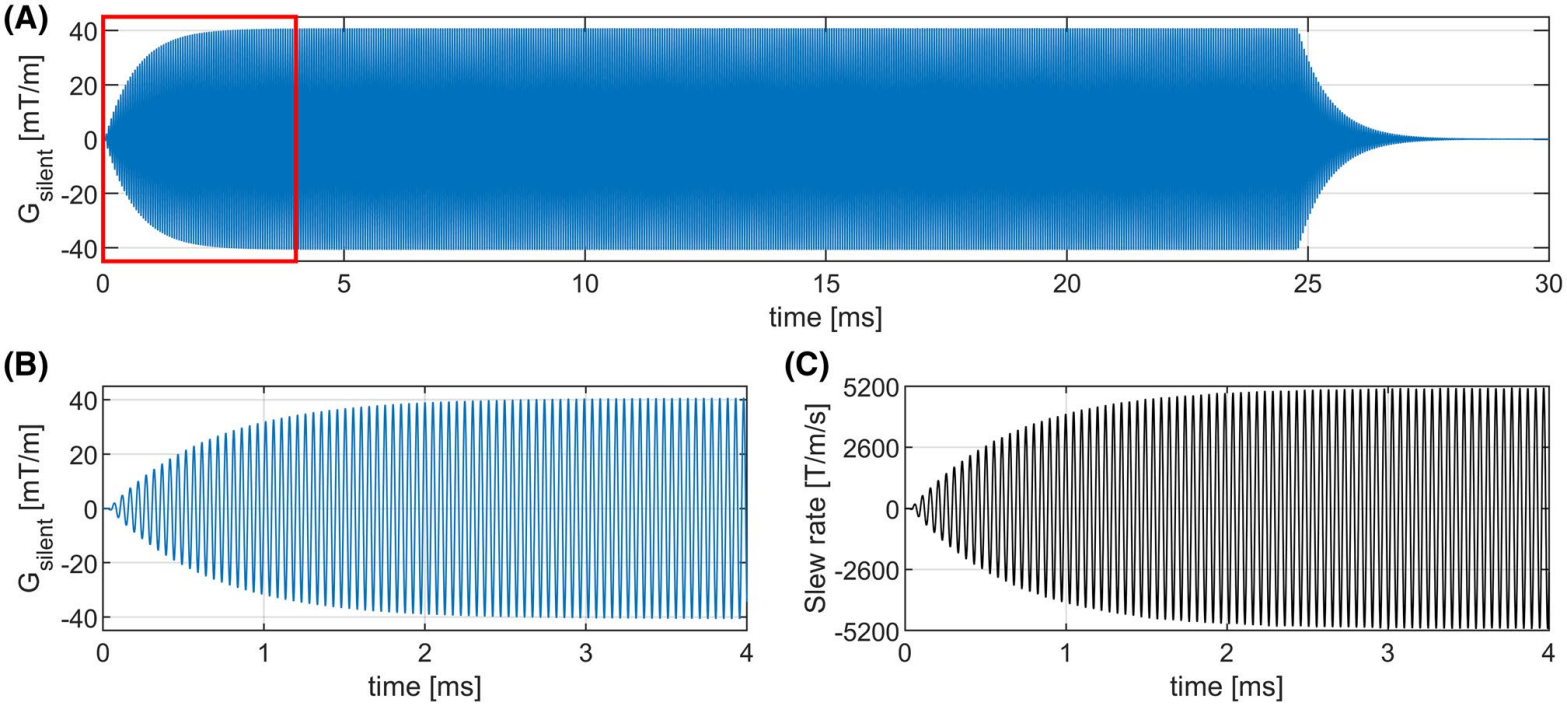
Results of the field measurement of the silent imaging setup. A, The gradient field measured during a 24 ms pulse train, showing a distinct startup‐period, steady‐state period and decay period. B, Detailed view of the startup‐phase of the silent gradient axis, showing the gradient field oscillating at 20.2 kHz and reaching a steady‐state amplitude of 40.8 mT/m. C, Detailed view of the measured slew rate, featuring a maximum slew rate of 5195 T/m/s

Figure 3A shows the full 24 ms pulse train with the gradient field oscillating at 20 kHz. Here, the resonant nature of the gradient coils resulted in three distinct periods: a startup‐period, a steady‐state and a decay‐period. Physically, the startup period represents a situation where the supplied power from the amplifier is higher than the resistive losses in the gradient coil, while the decay period represented the dissipation of energy in the coil after the pulsing has stopped. In the steady‐state, the losses in the gradient coil and supplied power are balanced. The startup period (Figure 3B) and decay period had a duration of approximately 3 ms. During the steady‐state, a maximum amplitude of 40.8 mT/m was measured, which translated to an encoded resolution of 3.65 cm in a half period of the oscillation. This amplitude corresponded to a peak field excursion of B_peak‐peak_ = 11 mT in the head/neck region. The peak slew rate during pulsing (Figure ) was 5178 T/m/s which is about 26‐fold higher than the 200 T/m/s that is used in whole‐body gradients.

### 3.2 Physiological effects

**FIGURE 4:**
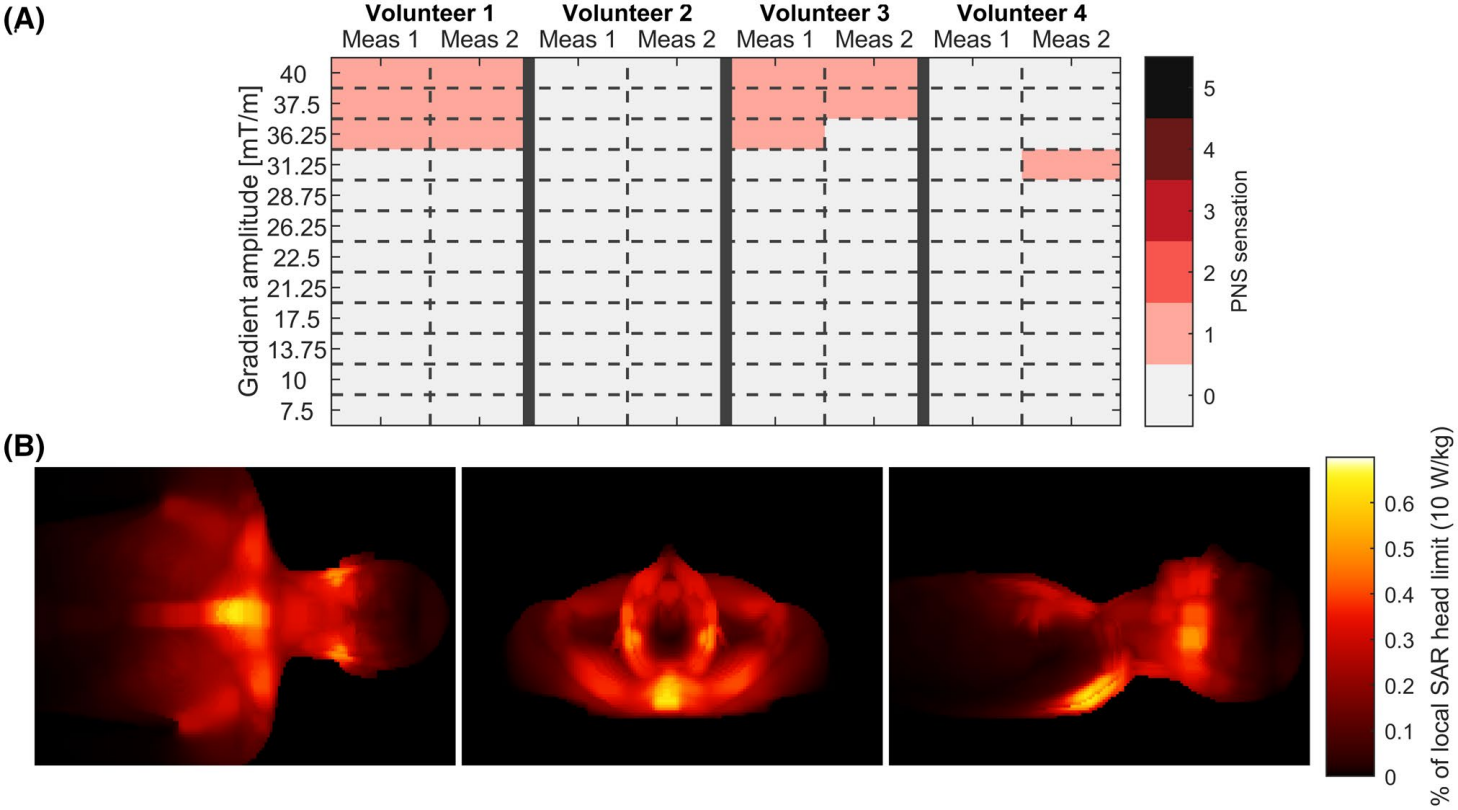
A, Results of the peripheral nerve stimulation measurements. Here, the PNS sensation scores were defined as: (0) no PNS, (1) very mild PNS, (2) mild PNS, (3) moderate PNS, (4) uncomfortable PNS and (5) painful PNS. B, Maximum intensity projections of SAR‐simulations with from left to right: coronal, transverse and sagittal projections

A summary of the PNS measurements is shown in Figure 4A. Here, two volunteers reported very mild PNS at gradient amplitudes and slew rates over 35 mT/m and 4422 T/m/s, respectively. Here, the PNS sensation was described as a light tingling feeling around the teeth or temple. The other two volunteers did not report any PNS at the highest slew rate. One volunteer reported a single occurrence of very mild PNS in the back of the head at 31.25 mT/m. However, this could not be replicated with an additional repetition of the measurement.

Figure 4B shows maximum intensity projections of the local SAR obtained from the SAR‐simulations. Here, the maximum local SAR was found to be 0.06 W/kg or 0.6% of the local SAR head limit. SAR hotspots were mainly located in the upper back and mouth area, which points to high electric fields in these areas.

**FIGURE 5:**
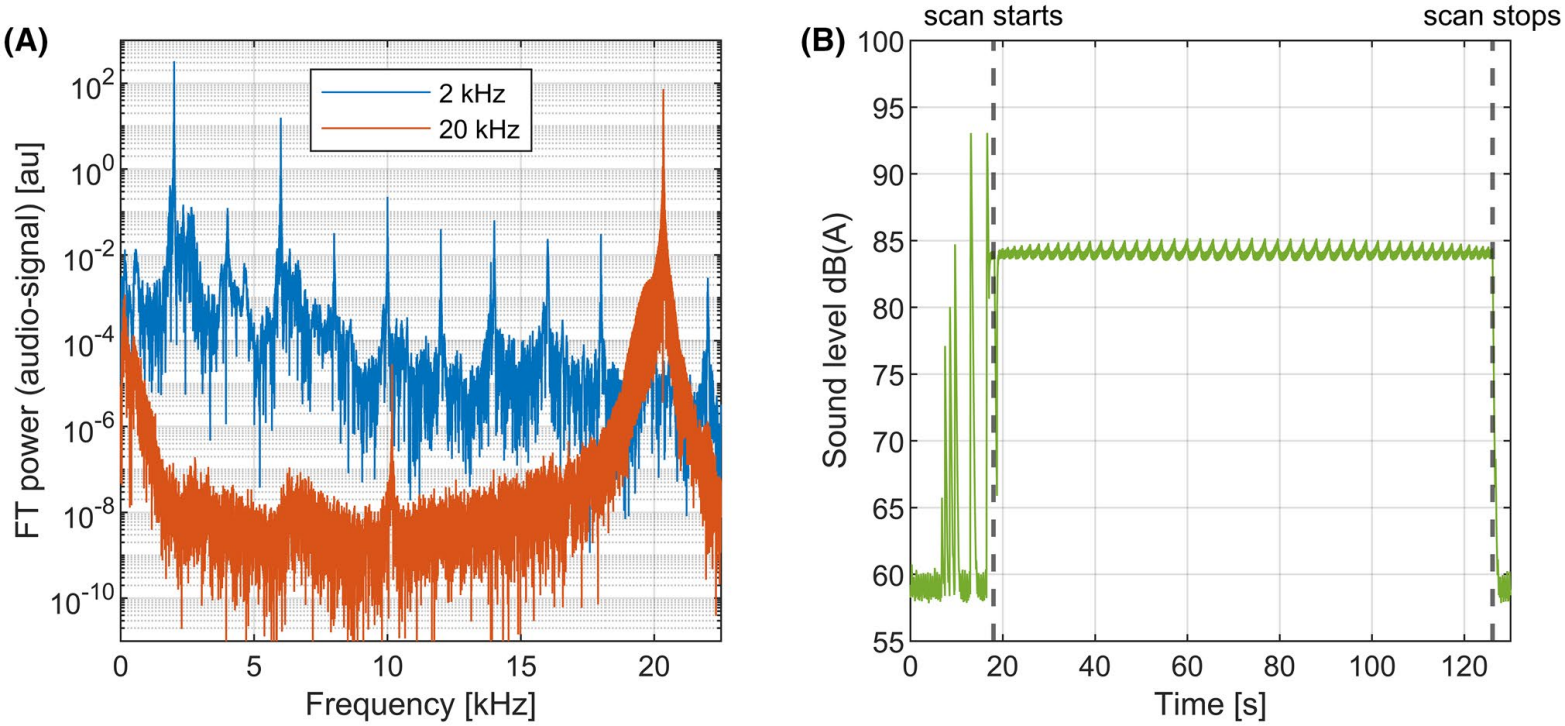
Results of the sound measurements on the silent gradient axis: A, Power spectra of the gradient insert driven at 30 mT/m at 2 and 20 kHz when the microphone is placed in the gradient insert. B, A‐weighted sound level during the 3D‐GRE acquisition made with the silent gradient axis

The acoustic power‐spectra of the 100 ms pulse‐trains of the gradient insert driven at 30 mT/m at 2 kHz and 20 kHz are shown in Figure 5A. Here, the acoustic signal power at 2 kHz was around 4.5 times higher than at 20 kHz. Furthermore, additional sound was produced at harmonic overtones when driven at 2 kHz. Almost no sound in the audible range was produced when the gradient insert was driven at 20 kHz. Here, the peak seen at 10 kHz was measured to be around 6x10^4^ times lower than the fundamental frequency of 20 kHz and could not be heard during the measurement. Figure 5B shows the sound level before and during the 3D GRE‐imaging scan. Here, a sound level of 85 dB(A) was measured during the 3D GRE‐imaging scan. The sound manifested itself as a low‐frequency humming sound during the scan which originated from the switching whole‐body gradients.

### 3.3 Imaging experiments

**FIGURE 6:**
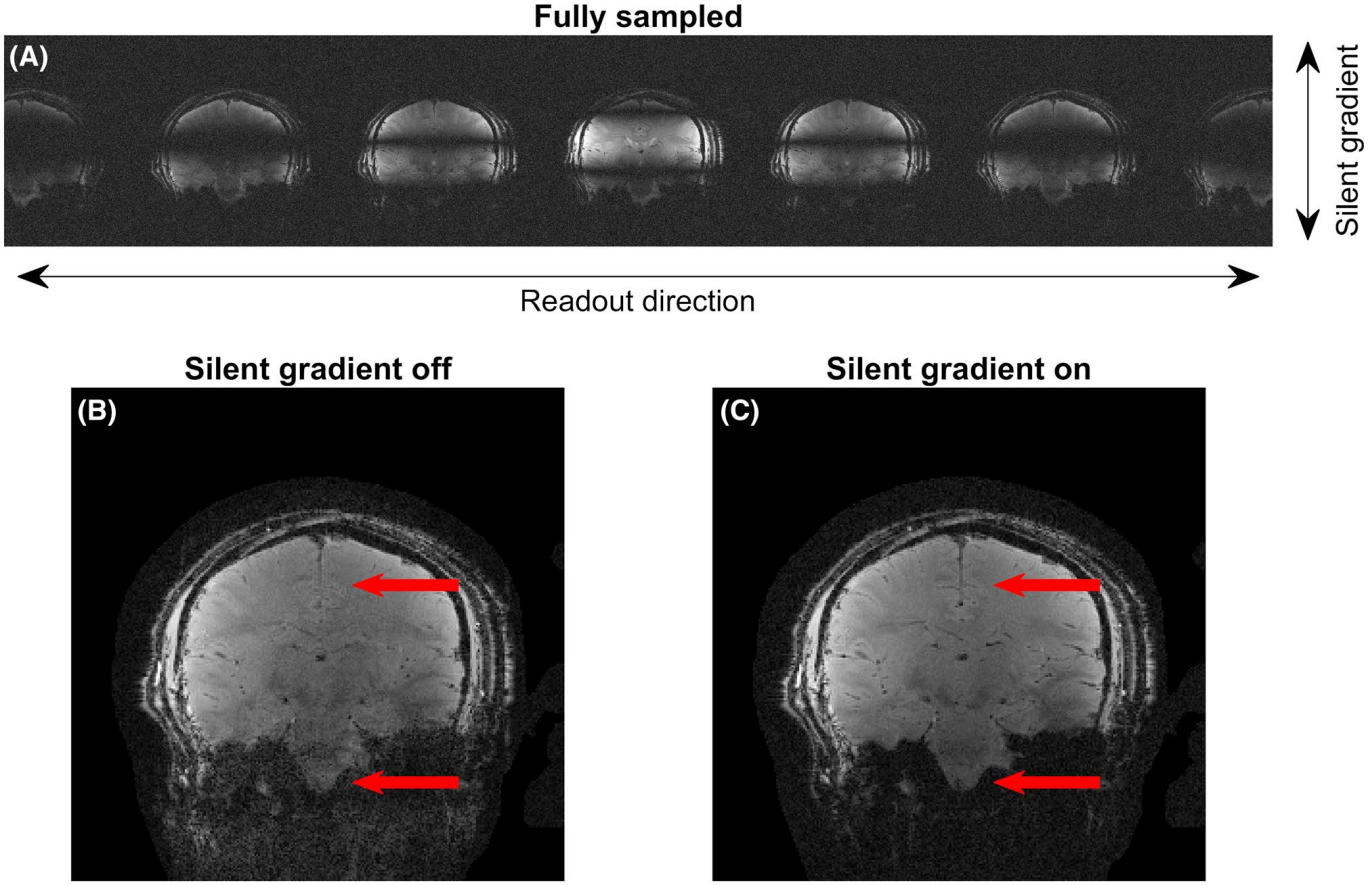
Results of 2D imaging experiments. A, the effect of the silent gradient axis on a fully sampled acquisition. Here, the silent gradient axis causes spreading (repetition) of the signal into the oversampled readout direction (the image is cropped to six‐fold oversampling). B, Image acquired with the silent gradient axis off but with larger phase‐encoding steps equaling Δ*k*_silent_. C, The arrows highlight the increased image noise (bottom arrow) and artifacts (top arrow) image acquired with the silent gradient axis driven at 31.5 mT/m

Figure 6 shows the effect of the silent gradient axis on a fully sampled 2D acquisition and 2D imaging results using our silent imaging method. When applied during a fully sampled acquisition (Figure 6A), the silent gradient axis caused a spreading (repetition) of the voxels in the readout direction (RL in this case) similar to wave‐CAIPI and bunched phase‐encoding. In Figure 6B,C, larger phase‐encoding steps were used that were determined using Equation (3). Here, the gradient amplitude of 31.5 mT/m yielded phase encoding steps of Δ*k*_silent_ = 21 m^ȡ21^ or 5.3 times Δ*k*_fully‐sampled_. In Figure 6B, these larger phase‐encoding steps were used without enabling the silent gradient axis, which resulted in a noisy image featuring aliasing artefacts. By enabling the silent gradient axis (Figure 6C), the noise in the image was reduced and aliasing artefacts were removed without increasing audible sound during the sequence.

**FIGURE 7:**
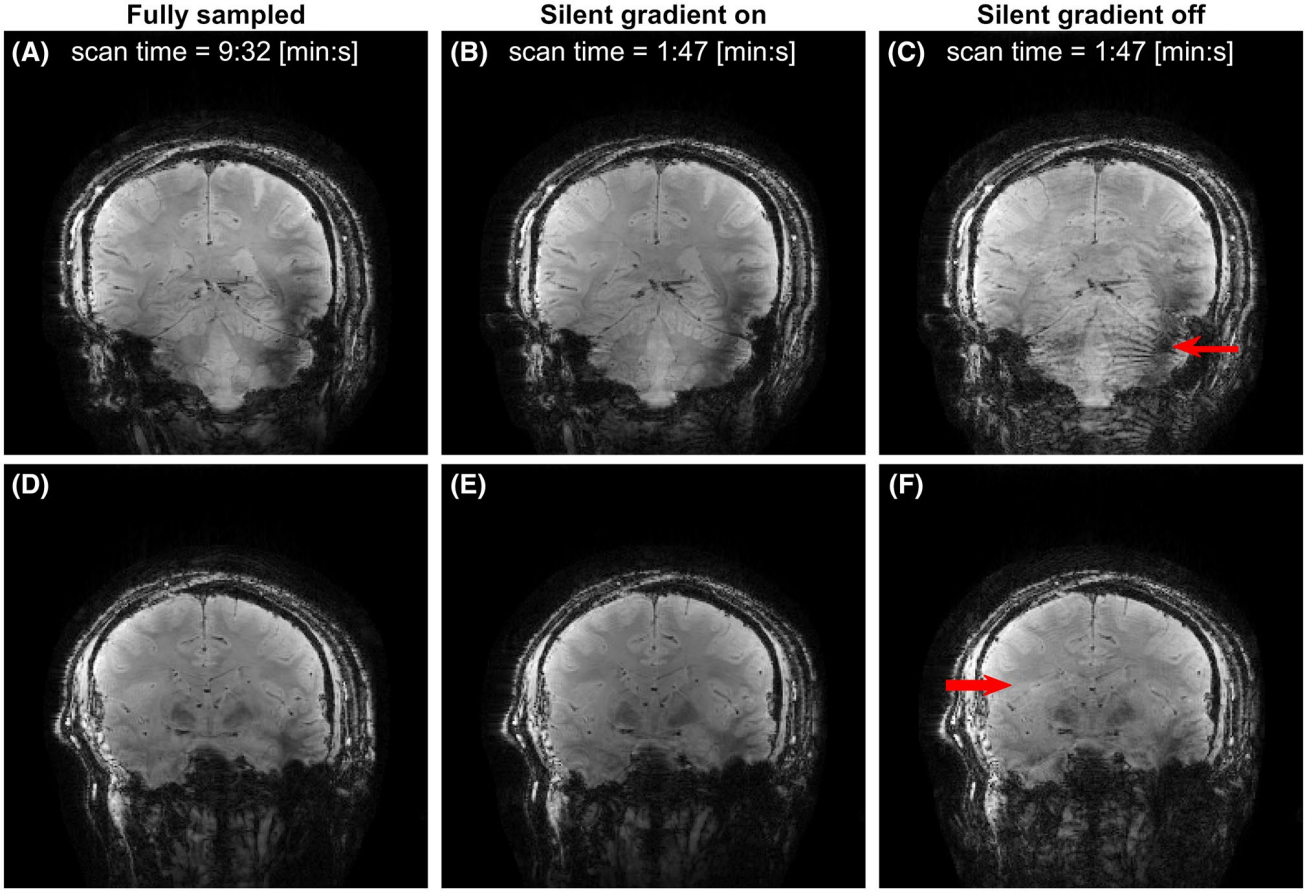
Representative slices of the 3D imaging experiments. A,D, images from the fully sampled acquisition with the silent gradient axis off. B,E, Images from the acquisition with the silent gradient axis driven at 31.5 mT/m. C,F, Images acquired with the silent gradient axis off but with larger phase‐encoding steps equaling Δ*k*_silent_. The red arrows indicate examples of artifacts not seen in the other acquisitions

Figure 7 shows the effect that the use of the silent gradient axis can have on both scan time and image quality. Figure 7B,E shows that our 3D silent imaging method featured no aliasing artifacts while being 5.3‐fold faster than the fully sampled acquisition (Figure 7A,D). Repeating the same acquisition without silent gradient yielded increased noise and aliasing artefacts, as can be seen in Figure 7C,F. Important to note is that the volunteer did not report any peripheral nerve stimulation when the silent gradient axis was driven.

## 4 DISCUSSION AND CONCLUSIONS

We have demonstrated the feasibility of a novel silent imaging method which uses a soundless spatial encoding axis to reduce audible sound while maintaining imaging speed. By greatly reducing the sound of an MRI sequence, our silent imaging method can substantially increase the subject comfort during an MRI examination. Increasing subject comfort and stimulating stillness during MRI will benefit patient groups in which sound is a contraindication, such as anxiety‐prone patients but also neurologically impaired, elderly and pediatric patients. In general, the increased MRI comfort with our silent imaging method is expected to greatly increase subject compliance in MRI, not only for patients but also for studies in healthy subjects, such as children. Increased compliance is expected to lead to decreased subject motion, which yields better image quality, fewer re‐scans and, thus, decreases scan time.^22^ Additionally, silent imaging is also of interest for neuroscience research as silent spatial encoding enables functional MRI studies without the confounding acoustic noise of the MRI exam.^23^

The field measurements demonstrated that the combination of a resonant gradient insert and an audio amplifier produced a gradient amplitude and slew rate of 40 mT/m and 5178 T/m/s, respectively. At this gradient amplitude and slew rate, peripheral nerve stimulation, tissue heating and sound do not seem to limit our silent imaging method. Half of the volunteers reported very mild PNS, but a more powerful amplifier and a larger sample size are needed to allow for an accurate estimation of the PNS threshold. In magnetic particle imaging, the body PNS threshold at a comparable switching rate (25 kHz) has been estimated to occur around a field excursion of B_peak‐peak_ = 15 mT.^12^, ^30^ Our silent gradient axis produced B_peak‐peak_ = 11 mT suggesting that higher silent gradient amplitudes should be physiologically possible, as the PNS threshold is expected to be lower for the head than the body. Moreover, when driven at a 100% duty cycle, tissue heating was found to be factor 167 (0.06 W/kg) lower than the local SAR head limit (10 W/kg), which means that PNS and not SAR will be limiting the maximum gradient performance at 20 kHz.

The audible and inaudible sound produced by the silent gradient axis were measured at the same gradient amplitude (30 mT/m), which ensured that the same peak forces were present in both measurements. Here, the silent gradient axis produced less sound at 20 kHz than at an audible frequency of 2 kHz, which we attributed to a lower acoustic response of the gradient insert at 20 kHz. The sound levels produced at 20 kHz are still in the same range of sound pressure levels (90–120 dB) that subjects are currently exposed to during MRI‐scans, which means that hearing protection is still needed. In literature, the reported main effects of low‐frequency ultrasonic sound (i.e., 20 kHz) are subjective, eg, headache, fatigue and nausea, also for sound levels lower than 120 dB.^33^, ^34^, ^39^ In this work, no such subjective effects were reported during all the PNS and imaging experiments. During the 3D imaging experiments, a sound level of 85 dB(A) was measured which was achieved by lowering the slew rates of the whole‐body gradients. A further reduction in sound level can be achieved by combining our approach with soft gradient pulses to reduce the contribution of high frequency components.^4^ Furthermore, our silent imaging method should also benefit from moving to a lower magnetic field strength (3T/1.5T) due to the intrinsically lower Lorentz forces.

Imaging experiments showed that artifact free images could be obtained for both 2D and 3D GRE sequences. Here, the extra spatial encoding provided by the silent gradient axis allowed us to acquire fewer phase‐encoding steps without introducing image artifacts. In this work, we used this increase in imaging efficiency to slow down the other encoding axes and reduce sound. Alternatively, the silent gradient axis could potentially be used for image acceleration by exploiting this silent spatial encoding to increase imaging efficiency without adding sound. We also showed that the silent gradient introduces voxel spreading in the readout direction, which has been shown in wave‐CAIPI and bunched phase‐encoding to lower g‐factor noise by exploiting coil sensitivity variations in three dimensions.^35^, ^36^ Additionally, our silent imaging method could also be combined with a point spread function based reconstruction which would allow for a inclusion of gradient field non‐linearities in reconstruction.^40^

The performance of the silent gradient axis was currently limited by the audio amplifier, as a lower gradient amplitude of 31.5 mT/m had to be used during the imaging experiments to prevent overheating of the amplifier. A possible solution to increase gradient performance and duty cycle at 20 kHz would involve significant modifications to a conventional PWM‐amplifier. To allow for operation at 20 kHz, both the switching behavior of the power stages and output filter characteristics would need be changed, which would require both major software and hardware modifications to the amplifier. Importantly, the silent gradient axis was limited to head‐only imaging, which is a limitation not present in other silent imaging methods like BURST, PETRA, and Looping Star.^6^, ^41^, ^42^ Translation of our silent imaging method to body imaging would require the use of local gradient coils suitable for body‐imaging or non‐linear gradient fields to limit PNS.^43^, ^44^

Our silent imaging method operates in synergy with the conventional encoding gradients and therefore is expected to be widely applicable as it requires minimal changes to existing cartesian sequences. Potential applications of our silent imaging method include (but are not limited to) clinically relevant sequences such as T1‐weighted and T2‐weighted anatomical imaging, and susceptibility weighted imaging. Here, we foresee two main challenges in applying our silent imaging method to these sequences. First of all, the sound reduction of our method relies on slower gradient switching and, thus, results in a longer repetition and echo time. Consequently, the feasibility of applying our silent imaging method to these sequences depends whether image contrast and SNR can be maintained at a clinically suitable level using these different imaging parameters. The second challenge is the single encoding axis of our silent gradient axis, which makes only sagittal and coronal slices possible for protocols using 2D‐imaging. However, when applied to 3D imaging no such limitation exists as the silent gradient axis can be applied in either phase or slice encoding direction. Moreover, the gradient insert used in this work was not explicitly designed to operate at 20 kHz. Therefore, the design of the gradient insert can also be improved to increase the available gradient performance at 20 kHz by optimizing the efficiency at the resonant frequency and by incorporating PNS constraints in the design process.^45^ Furthermore, we expect that the silent gradient concept presented here can be applied to the other gradient axes (*X* or *Y*). Note that compared to a *Z*‐axis gradient, transverse *X*/*Y*‐axis gradient designs feature intrinsically lower gradient efficiency and PNS thresholds, which results in higher power requirements and a slightly lower maximum gradient amplitude. However, increases in imaging efficiency are still expected, especially when combining multiple silent gradient axes.

In conclusion, we have presented a novel silent imaging method that can reduce sound without compromising scan time by using a resonant gradient axis driven at 20 kHz consisting of a gradient insert and audio amplifier for spatial encoding. Using this setup, physiological effects such as PNS were found to not limit the operation despite the high slew rate (5178T/m/s) while artifact free images were obtained for both 2D and 3D GRE sequences featuring low acoustic noise.
